# Editorial: Health services and the 4th industrial revolution

**DOI:** 10.3389/frhs.2025.1646779

**Published:** 2025-07-09

**Authors:** Joris Van De Klundert, Michal Mankowski, Harwin De Vries

**Affiliations:** ^1^ Business School, Adolfo Ibáñez University, Santiago, Chile; ^2^ Department of Surgery, Grossman School of Medicine, New York University, New York, NY, United States; ^3^ Department of Technology & Operations, Management, Rotterdam School of Management, Erasmus Universiteit Rotterdam, Rotterdam, Netherlands

**Keywords:** 4th industrial revolution, health services, technology acceptance, stakeholder engagement, SDG3: good health and wellbeing

**Editorial on the Research Topic**
Health services and the 4th industrial revolution

The 4th industrial revolution (4IR) advances and its impact is being felt far beyond the manufacturing sector that is commonly associated with former industrial revolutions (1). The first two industrial revolutions have automated the manufacturing, energy and transportation sectors, developments which have enabled the growth of the service sectors (2). More advanced economies tend to have larger service sectors which tend to be more labor intensive and therefore employ an even larger part of the workforce (3, 4). The health services sector is one of the service sectors that has witnessed especially significant growth. In the US, for instance, it contributes around 17 percent to GDP—almost as much as all non-service sectors together—and contributes even more to employment (4–6). Globally, health expenditure has recently risen above 10 percent of the global GDP (7).

The 4IR has been defined as “the advent of ‘cyber-physical systems’ which involve entirely new capabilities for people and machines” (8). Through novel technologies which enable to blend “physical, digital, and biological changes” it is predicted to “fundamentally alter the way we live, work, and relate to one another” (9). This blend especially opens new venues for the health services “industry” which naturally combines these three domains.

This research topic “*Health services and the 4th industrial revolution*” explores scientific and practical understanding of the disruptive impact of the 4th industrial revolution on health services. The contributions made address scientific and practical developments already taking place and reflect on current and future implications. They vary from a reflection on the place of the health service industry in the technology dynamics, to a practice and policy brief on digital health, original research on the use of black box analytics, and methodological considerations for mHealth. The contributions range from addressing adoption of 4IR technologies in well-resourced settings such as the USA to implementation in resource constraint, low income, settings.

Zhai convincingly argues that “while the semiconductor industry could remain profitable without the health sector, the health sector could not exist in its current form without the semiconductor industry”. This highlights the vulnerability of the health services sector and the dependence on priorities set by a small number of dominant players in other dynamic industries driving the 4th industrial revolution. These vulnerabilities may be exacerbated by global political developments in the years to come.

Rinke de Wit et al. also depart from a vulnerability, in particular the vulnerability of fragile health systems, as exposed by the Covid-19 pandemic when supply chain priorities favored high income countries. This exacerbated the pandemic response difficulties experienced in low- and middle-income countries. On the positive side, the authors describe the adoption of novel information technologies that emerged in this challenging context, referred to as “digital health systems strengthening”. They show how effective digital interventions can effectively engage relevant stakeholders from patients to policy makers and providers and contribute to strengthening the health service systems in Africa and achieving SDG3, i.e., to “ensure healthy lives and promote well-being for all at all ages” (10).

This global development goal and the persistent shortages in the funding, workforce, and other necessary resources are also the starting point for van de Klundert et al. They explore whether new technologies, as brought along by the 4th industrial revolution, in particular advanced predictive and prescriptive analytics, can help address resource shortages to promote the effectiveness and equity of health services. More specifically, they zoom in on the question of whether the more advanced “black box” models, despite being less explainable, can improver the effectiveness-equity frontier. The results of two case studies, one from a high income setting and one from a low income setting, suggest that the more advanced and less explainable models provide little additional benefits (if at all) that justify the drawbacks various stakeholders associate with technologies that are lacking in explainability (11).

Hankins et al. also consider a high income setting. They address methodological considerations for the evaluation of a multi-stakeholder mHealth implementation to promote adherence to evidence based sickle cell disease medication. The approach again emphasizes the importance of involving multiple stakeholders from multiple levels and to adequately address behavioral aspects of technology adoption and implementation.

All contributions to the research topic emphasize the inclusion of all stakeholders in the supply chains and value networks of health service provisioning as a prerequisite for successful adoption of 4IR technologies and subsequent impact on health and well-being. In part, this engagement can be achieved based on current evidence and theories from health service innovation and implementation science such as the technology acceptance model and its successors (Hankins et al., 12). In addition, the new technologies bring along new factors such as explainability and interpretability that strongly interact with professional and ethical values and need to be incorporated in health service innovation models for the 4th industrial revolution (van de Klundert et al., 13).

Likewise, the contributions reveal that industry and policy dynamics might easily cause the benefits of the new technologies to be inequitably distributed, as salient stakeholders prioritize other objectives over SDG3 or as unintended side effects of novel—black box—technology adoption (Zhai, Rinke de Wit et al.). The speed and pervasive impact of the unfolding 4th industrial revolution necessitate practice and research to adhere to the highest ethical standards to ensure that benefits preferentially reach those most in need.
